# From Basics to Breakthroughs: A Review on the Evolution of *Campylobacter* spp. Culture Media

**DOI:** 10.3390/microorganisms14020498

**Published:** 2026-02-19

**Authors:** Ana Rita Barata, Maria José Saavedra, Gonçalo Almeida

**Affiliations:** 1 National Institute of Agricultural and Veterinary Research (INIAV), 4485-655 Vila do Conde, Portugal; anarita.barata@iniav.pt; 2 Centre for the Research and Technology of Agro-Environmental and Biological Sciences (CITAB), Institute for Innovation, Capacity Building and Sustainability of Agri-Food Production (Inov4Agro), University of Trás-os-Montes and Alto Douro, 5001-801 Vila Real, Portugal; saavedra@utad.pt; 3 Center for Animal Science Studies (CECA-ICETA), Associated Laboratory of Animal and Veterinary Science (AL4AnimalS), University of Porto, 4099-002 Porto, Portugal

**Keywords:** selective media, culture methods, microaerophilic growth, food microbiology, diagnostic innovation, food pathogens

## Abstract

Since their recognition as human pathogens in the 1970s, *Campylobacter* spp. have posed persistent challenges to microbiologists due to their fastidious growth requirements and environmental sensitivity. The continuous refinement of selective and differential culture media has been crucial for improving their detection, isolation, and characterization in both clinical and food microbiology. This comprehensive review provides a chronological overview of the evolution of *Campylobacter* culture media, highlighting the scientific milestones that shaped current cultivation practices—from early blood- and charcoal-based formulations to modern selective, chromogenic, and systems permitting incubation under less stringent atmospheric conditions. Emphasis is placed on the rationale behind medium composition, the transition from empirical experimentation to standardized formulations, and the integration of molecular and metabolic insights into media design. The evolution of *Campylobacter* growth media mirrors the broader trajectory of microbiology itself, moving from artisanal experimentation toward precision-driven innovation. Ongoing advancements in culture technology, including sustainable and data-guided formulations, will continue to enhance global surveillance, food safety, and pathogen ecology research.

## 1. Introduction

*Campylobacter* spp., particularly *Campylobacter jejuni* and *Campylobacter coli*, are among the leading causes of bacterial gastroenteritis worldwide, representing a major concern for public health and food safety [1]. Campylobacteriosis has been the most frequently reported zoonotic disease in the European Union since 2005, consistently surpassing other zoonoses such as salmonellosis, while also being recognized as one of the leading causes of bacterial gastroenteritis worldwide, with a substantial disease burden reported in North America, Asia, Oceania, and parts of Africa [2,3,4]. Its widespread prevalence is largely associated with poultry consumption and foodborne transmission, reinforcing the importance of *Campylobacter* as a major public health and food safety concern. This trend continues to be confirmed by the most recent European Food Safety Authority (EFSA) report, which identifies *Campylobacter* as the leading zoonotic agent in 2024 [5]. The ability to accurately detect, isolate, and study these microorganisms is pivotal for understanding their epidemiology, pathogenesis, and antibiotic resistance patterns [6]. Central to these efforts is the development and refinement of culture-based methodologies, specifically the growth media designed to support the unique physiological requirements of *Campylobacter* species [7]. *C. jejuni* and *C. coli* are responsible for the vast majority of reported campylobacteriosis cases, and culture-based detection methods have therefore been historically developed with a strong focus on these thermotolerant species [8]. In the early stages of *Campylobacter* diagnostics, incubation at 37 °C was commonly applied, largely by analogy with other enteric pathogens and reflecting the recent recognition of *Campylobacter* as a human pathogen [9]. Subsequent studies in the late 1970s and early 1980s demonstrated that *C. jejuni* and *C. coli* grow optimally at approximately 42 °C, a temperature that mirrors the intestinal environment of birds, their primary reservoir [10,11]. The transition to incubation at 42 °C improved recovery and selectivity by enhancing growth of these species while suppressing competing flora, leading to its adoption in selective media and standardized methods [12]. However, this optimization also introduced a methodological bias, as non-thermotolerant *Campylobacter* species, such as *Campylobacter lari* and *C.ampylobacter upsaliensis*, are less efficiently recovered under these conditions and may therefore be under-detected.

Historically, the isolation and cultivation of *Campylobacter* spp. presented a considerable challenge to microbiologists, attributed to their intricate growth requirements and the lack of suitable culture media. Early efforts to culture these organisms often resulted in low recovery rates and poor growth. However, the ensuing decades have witnessed significant advancements in the formulation of growth media, driven by a deeper understanding of the microbial physiology of *Campylobacter* and the innovative application of molecular biology techniques [13,14].

This review article aims to trace the evolution of growth media for *Campylobacter* spp., from the initial attempts at isolation to the sophisticated, selective media available today. By examining the chronological development and scientific rationale behind various media formulations, we seek to highlight the milestones in the cultivation of these important pathogens. Through a comprehensive analysis of the literature, this article will identify emerging trends in media development and suggest future directions for research. In doing so, we aim to provide a valuable resource for microbiologists, food safety experts, and researchers dedicated to the study of *Campylobacter* spp., facilitating further innovation in this critical area of microbiology.

## 2. The Genus *Campylobacter*

*Campylobacter* comprises a diverse group of Gram-negative, curved bacteria within the family *Campylobacteraceae*, including more than 30 recognized species. *Campylobacter jejuni* and *C. coli* account for most human infections, whereas species such as *C. lari*, *C. upsaliensis*, *Campylobacter fetus*, and *Campylobacter hepaticus* are mainly associated with animal hosts but retain zoonotic relevance [15]. Variation in host adaptation, temperature preference, and environmental tolerance contributes to differences in pathogenic potential and recovery efficiency during laboratory culture [2].

These bacteria are metabolically specialized, chemoorganotrophic organisms that are generally nonproteolytic, nonlipolytic, and nonsaccharolytic. They derive energy primarily from the oxidation of amino acids and tricarboxylic acid cycle intermediates rather than from carbohydrate fermentation or oxidation. Their fastidious physiology requires reduced oxygen tension and elevated carbon dioxide concentrations for optimal growth. Many pathogenic species are thermotolerant, reflecting adaptation to avian reservoirs, and exhibit marked sensitivity to oxidative stress and inhibitory compounds, necessitating protective growth environments—an important factor that has strongly influenced the formulation of selective culture media [16].

Ecologically, *Campylobacter* species frequently inhabit the gastrointestinal tract of a broad range of hosts, including poultry, livestock, companion animals, and wildlife, where colonization is often asymptomatic. Their widespread presence in animal reservoirs and contaminated food underscores their zoonotic transmission potential and central role in foodborne disease [17]. From a clinical and ecological perspective, species within the genus can be broadly grouped into thermotolerant strains of primary public health importance, livestock-associated species that infrequently cause human disease, taxa linked to specific human conditions such as periodontal infections, and environmental or host-associated species not currently implicated in foodborne illness. This diversity reflects the adaptive capacity of the genus and contributes to variability in host range, virulence traits, and epidemiological significance [18].

At the genomic level, *Campylobacter* displays considerable plasticity that supports adaptation to diverse ecological niches and influences virulence, antimicrobial resistance, and metabolic capacity. Motility, adhesion, and toxin-associated functions are tightly regulated in response to environmental cues, shaping bacterial behavior during host colonization and laboratory cultivation [19]. Collectively, these biological traits underpin the demanding growth requirements of *Campylobacter* spp. and provide essential context for understanding the development of selective media designed to balance organism recovery with suppression of competing microbiota.

## 3. Literature Overview

This review provides an integrative and chronological overview of the development of culture media for *Campylobacter* spp. from the late 1960s to the present day. Relevant literature was identified primarily through searches in scientific databases such as PubMed, Scopus, and Web of Science, complemented by reference tracking of key publications and international microbiological standards.

Priority was given to peer-reviewed studies describing the formulation, optimization, or comparative evaluation of selective and enrichment media used for *Campylobacter* isolation in food, clinical, and environmental contexts. Foundational reports and historical milestones that contributed to the conceptual or technical evolution of *Campylobacter* cultivation were also included to ensure a coherent representation of the field’s progression.

This narrative and interpretative approach aims to synthesize the main scientific trends, methodological advances, and practical innovations rather than exhaustively list all available studies. Emphasis is placed on identifying the rationale behind medium composition, the transition from empirical to standardized formulations, and the impact of these developments on diagnostic and food safety practices.

In addition to primary research articles, selected review papers, ISO guidelines, and reference protocols were consulted to contextualize major shifts in the understanding of *Campylobacter* physiology and its implications for culture medium design.

## 4. Evolution of *Campylobacter* Culture Media

The development of culture media for *Campylobacter* spp. represents a relatively recent phase in the history of microbiology. Reliable methods for isolating these organisms only began to emerge in the early 1970s, largely due to their fastidious growth requirements and pronounced sensitivity to environmental conditions. This delayed methodological progress contrasts sharply with that of other major foodborne pathogens, such as *Listeria monocytogenes*, which was isolated and scientifically described several decades earlier [20], and *Escherichia coli*, first described by Escherich in the late nineteenth century [21]. Consequently, *Campylobacter* remained underrecognized as a human and animal pathogen for much of the twentieth century.

Collectively, these studies illustrate how advances in *Campylobacter* culture media have been shaped by the evolving requirements of clinical diagnostics, veterinary surveillance, and food safety monitoring. The following sections present a chronological overview of these developments, highlighting the key scientific and technical milestones that led from early empirical approaches to the standardized and specialized media used today.

### 4.1. Pioneering Attempts (1970s)

Although *Campylobacter* spp. were not recognized as human pathogens until the 1970s, historical records indicate that these organisms had likely caused human and animal illness for decades. In 1886, Escherich described spiral-shaped bacteria in children who had died from “cholera infantum,” but he was unable to culture them successfully [22]. Similar organisms were later observed in sheep by McFadyean and Stockman (1909) [23], and isolated from cattle by Smith (1919), who named the organism *Vibrio fetus* [24,25]. Throughout the following decades, its pathogenic relevance in livestock became increasingly evident [26,27].

By the 1930s and 1940s, additional links between *Vibrio*-like organisms and enteric diseases were documented in calves, swine and, occasionally, humans [28,29,30]. King’s work in 1957 marked another important milestone, as he identified a “related *vibrio*” with unique physiological traits that hindered routine cultivation using existing laboratory methods [31]. Although King attempted to adapt fecal culture techniques, the absence of selective media and the organism’s fragile physiology resulted in poor recovery, contributing to its under-recognition in clinical microbiology.

A critical conceptual shift occurred when Sebald and Véron (1963) used DNA base composition to reclassify these vibrios under the new genus *Campylobacter* [32]. However, despite clearer taxonomic identity, isolation remained unreliable. Until the late 1960s, most attempts to culture *Campylobacter* spp. yielded inconsistent or non-viable growth, reflecting the need for selective media and improved atmospheric control.

In summary, early discoveries of *Campylobacter*-like organisms highlighted their clinical and veterinary relevance but also exposed the major technical barriers to their cultivation. The lack of appropriate media, inadequate incubation conditions, i.e., atmosphere and temperature systems, and reliance on empirical methods led to decades of underdiagnosis [10]. These foundational challenges ultimately set the stage for the first successful isolation from fecal samples in 1968–1972, marking the transition to the modern era of *Campylobacter* culture methods.

### 4.2. The Crucial Step

The turning point came in the late 1960s, when Dekeyser, in collaboration with Butzler and colleagues, developed the first reliable method for isolating *Campylobacter* directly from fecal material. This pivotal advance, achieved in 1968 and published in 1972, overcame many of the technical limitations that had hindered detection for nearly a century. They successfully isolated a ‘related vibrio’ (*Campylobacter*) from a patient’s blood and subsequently from feces using an innovative filtration technique. This method involved the differential filtration of fecal suspensions through filters with a pore size of 0.65 µm, enabling the passage of *Campylobacter* organisms. The filtrate was then applied to a selective growth medium (thioglycolate-agar medium supplemented with defibrinated sheep blood, bacitracin, polymyxin B sulfate, novobiocin and actidione) [33]. This landmark fecal culture confirmed that the source of the bacteremia was an intestinal infection.

In the following years, various authors [34,35,36,37,38,39] used the same methodology to isolate *Campylobacter* from bovine and sheep fetuses, human samples, and bull semen or preputial mucus, facing significant challenges and determining that numerous cultures failed to thrive in deep culture conditions.

In summary, the period from 1968 to the early 1970s marked the first reliable isolation of *Campylobacter* from fecal and clinical samples. Filtration-based methods [34,35] enabled critical early breakthroughs and broadened the recognized ecological and clinical range of the organism [36,37,38,39]. Yet, their technical complexity underscored the urgent need for more practical, selective culture media, setting the stage for the development of blood-based formulations in the late 1970s. As the limitations of filtration became increasingly clear, attention shifted toward developing selective blood-based media that offered greater reproducibility, simplified workflows, and improved recovery of thermophilic *Campylobacter*.

The early selective media summarized in Table 1 illustrate the empirical strategies initially employed to overcome the extreme fastidiousness of *Campylobacter* spp. Fluid thioglycolate-based formulations relied on strongly reduced environments, blood supplementation, and broad-spectrum antibiotics to permit survival during filtration-based isolation. The Butzler medium [40] represented an optimization rather than a conceptual departure from this approach, refining blood concentration and incubation conditions. In parallel, the VA-10 [35] formulations marked an early attempt to introduce more defined metabolic components, including succinate and iron salts, and to partially replace blood with hematin. Despite these innovations, all early media remained technically demanding, slow, and insufficiently selective, underscoring the need for simpler and more reproducible blood-based selective media developed in the late 1970s.

### 4.3. Blood-Based Selective Media (1977–1982)

The use of blood as a supplement in culture media was already well established in clinical microbiology for the recovery of fastidious and clinically relevant microorganisms, including *Haemophilus* spp. and *Neisseria* spp., as it provides essential growth factors, neutralizes inhibitory substances, and enhances bacterial growth and recovery [41].

In 1977, Skirrow [10] introduced a practical and highly influential method for isolating *Campylobacter* spp. from fecal samples, marking one of the most decisive advancements in *Campylobacter* diagnostics. He replaced the labor-intensive filtration step with a selective blood-based medium (Table 2). Incubation in a vacuum jar at approximately 43 °C suppressed competing enteric flora while supporting the growth of thermophilic *Campylobacter* species. Although Skirrow medium significantly improved selectivity and routine recoverability, the antibiotic combination introduced an inherent selection bias, inhibiting antibiotic-susceptible *Campylobacter* strains and reducing the recovery of non-*C. jejuni*/*C. coli* species. Building on this work, several research groups introduced blood-containing selective media that enhanced routine detection and eliminated the need for pre-enrichment or filtration [42,43,44,45,46,47]. These refinements focused on optimizing antibiotic combinations, stabilizing blood components, and improving growth performance across a wider range of sample types; however, their antibiotic composition and incubation conditions increasingly favored *C. jejuni* and *C. coli*, introducing an early species-level recovery bias. As these selective formulations became widely adopted, *Campylobacter* spp. rapidly gained recognition as common human pathogens, transitioning from previously underdiagnosed organisms to clinically significant agents of bacterial gastroenteritis.

The development of blood-based selective media during this period established the foundation for modern culture-based diagnostics (Table 2). These media enabled consistent recovery from fecal specimens, supported epidemiological investigations, and paved the way for further diversification, including the development of charcoal-based, antifungal-supplemented, and commercially formulated media suitable for food testing and automated laboratory workflows. Nevertheless, subsequent blood-based selective media, while enhancing the recovery of *Campylobacter* species, retained antibiotic-driven selection bias toward veterinary-associated *Campylobacter* species. Between 1977 and 1982, the introduction of selective blood-based media transformed *Campylobacter* detection from an experimental challenge into a routine laboratory procedure. Skirrow’s formulation offered the first practical alternative to filtration, and subsequent blood-containing media refined selectivity and reliability [42,43,44,45,46,47]. Lysed horse blood is incorporated into culture media for *Campylobacter* spp. because it provides essential growth factors and protection against inhibitory compounds. *Campylobacter* species are fastidious, microaerophilic organisms with limited capacity for de novo heme synthesis; therefore, erythrocyte lysis is required to release heme and hemin, which are critical for respiratory enzyme function and energy metabolism [48,49]. In addition, lysed blood supplies readily available iron and other cofactors and reduces oxidative stress by neutralizing reactive oxygen species and binding toxic metabolites such as free fatty acids and peroxides. Horse blood is preferred due to its consistent composition, lower variability, and reduced levels of inhibitory substances compared with other animal blood sources, and its use has been extensively validated in standardized media formulations for reliable recovery of *Campylobacter*, particularly from stressed samples [11].

**Table 2:** Selective media developed for the isolation of *Campylobacter* spp. between 1977 and 1982 were predominantly blood-containing culture media.

	Name	Basal Medium (Per Litter)	Supplements (Per Litter)	Refs.
Broth	Preston broth	Nutrient broth No. 2: peptone (10 g); meat extract (10 g); sodium chloride (5 g)	Actidione (0.0001 g) Polymyxin (0.005 IU) Rifampicin (0.0001 g) Saponin-lysed horse blood (50 g) Trimethoprim (0.0001 g)	[11]
	-	Brucella broth Cysteine hydrochloride (0.1g) Sodium succinate (3 g)	Cycloheximide (0.05 g) Lysed horse blood (70 g) Polymyxin B (20,000 IU) Trimethoprim (0.005 g) Vancomycin (0.015 g)	[50]
Agar	Skirrow medium (Campy agar)	Blood agar base: peptones (10 g); meat extract (5.0 g); sodium chloride (5.0 g); agar (12.0–15.0 g)	Lysed horse blood (50 g) Polymyxin B sulphate (25 IU) Trimetropim (0.05 g) Vancomycin (0.10 g)	[10]
	Preston agar	Same formulation as in Preston broth Agar (1–2%)	Same supplements as in Preston broth	[11]
	Campy-BAP	Brucella agar base: peptones (10 g); casein enzymic hydrolysate (10 g); yeast extract (2.0 g); dextrose (1.0 g); sodium chloride (5.0 g); agar (12.0–15.0 g)	Amphotericin B (2000 g) Polymyxin B (2.5 IU) Sheep red blood-cells (100 g) Trimethoprim (0.05 g) Vancomycin (0.01 g)	[51]
	-	Brucella agar base Sodium metabisulfite (0.25 g) Sodium pyruvate (0.25 g)	FBP supplement (0.25 g)	[52]
	modified Skirrow medium (mSK)	Agar base	Bacitracin (25 IU) Horse blood (50 g) Polymyxin B sulphate (2500 IU) Trimethoprim lactate (0.05 g)	[53]
	Campy-thio	Same formulation as in Butzler medium (Table 1)	Amphotericin B (0.02 g) Cephalothin (0.001 g) Polymyxin B (2.5 IU) Trimethoprim (0.05 g) Vancomycin (0.10 g)	[54]
	modified Butzler (BU40)	FTM-A (Table 1)	Actidione (0.5 g) Bacitracin (25,000 IU) Cephalothin (0.15 g) *Coli*stin (40,000 IU) Defibrinated sheep blood (100 g) Novobiocin (0.05 g)	[55]

**Figure 1:**
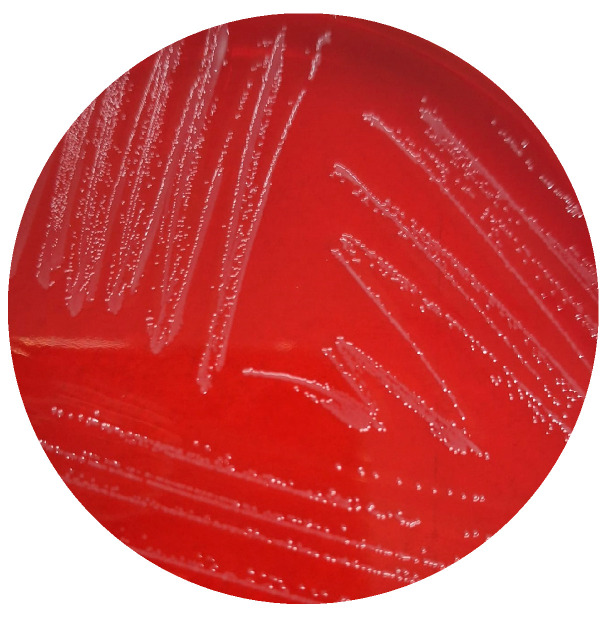
*Campylobacter coli* on blood agar (authors’ own photograph).

These advances not only confirmed *Campylobacter* as a major enteric pathogen but also laid the groundwork for the transition, during the 1980s, toward charcoal-based and fully blood-free media. Although blood-based media (Figure 1) were initially successful, they presented inherent limitations, including variability associated with animal-derived components and difficulties in colony visualization. These challenges ultimately drove the development of charcoal-based and completely blood-free formulations, marking the next major phase in the evolution of *Campylobacter* culture methods.

### 4.4. Charcoal-Based and Non-Blood Media (1983–1990)

The period between 1983 and 1990 marked a decisive turning point in the development of growth media for *Campylobacter* spp., characterized by the gradual replacement of blood with activated charcoal and reducing agents (Table 3). This shift aimed to eliminate biological variability, improve colony visibility, and reduce costs associated with animal-derived ingredients. Activated charcoal functions as a non-specific adsorbent of toxic metabolites, peroxides, and fatty acids. Bolton et al. (1983) demonstrated this effect by systematically comparing basal media with and without charcoal, showing that charcoal-containing formulations enhanced aerotolerance and supported *Campylobacter* growth under otherwise inhibitory oxygen conditions, indicating removal of oxygen-derived toxic compounds from the medium, thereby reducing oxidative stress and creating a protective microenvironment for oxygen-sensitive *Campylobacter* cells [56].

The first significant advancement came from Bolton et al. (1983) [56], who introduced a blood-free medium supplemented with bacteriological charcoal, ferrous sulfate, and sodium pyruvate, creating a reductive environment that protected oxygen-sensitive organisms. A year later, Bolton et al. (1984) [43] refined the formulation to produce the selective medium Charcoal Cefoperazone Deoxycholate Agar (CCDA) (Table 3), which effectively inhibits competing flora while promoting the growth of *C. jejuni* and *C. coli*.

**Figure 2:**
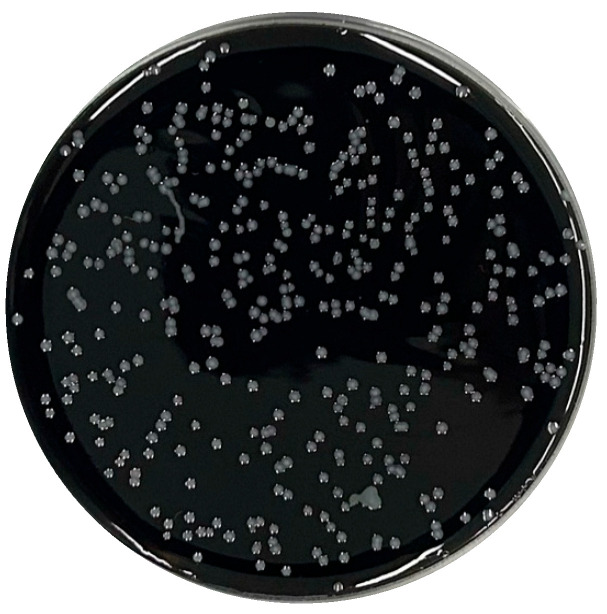
*Campylobacter jejuni* on mCCDA (authors’ own photograph).

Subsequently, Hutchinson and Bolton (1984) [57] modified this formulation into the mCCDA (Figure 2), standardizing the cefoperazone concentration. This version later became the international reference medium incorporated into ISO 10272-1:2017 Part 1 [58] and ISO 10272-2:2017 Part 2 [59] protocols for food and animal feeding stuffs testing. Parallel to these developments, other researchers explored enrichment and semi-solid formulations. Doyle and Roman (1982) [50] proposed an enrichment broth based on Brucella broth, cysteine, and lysed blood, allowing rapid growth under agitation at 42 °C. In turn, Goossens et al. (1989) [46] introduced a semi-solid, blood-free motility medium (SSM) that exploited *Campylobacter*’s characteristic motility, facilitating differentiation from competing fecal flora.

The introduction of charcoal-based and blood-free formulations represented a paradigm shift, eliminating reliance on blood-containing media, improving productivity and oxygen tolerance, and laying the groundwork for the global standardization of *Campylobacter* isolation. However, the widespread use of cefoperazone in these media preferentially favors intrinsically resistant *C. jejuni* and *C. coli*, thereby reinforcing species-level selection bias.

### 4.5. Rise in Antifungal and Commercial Formulations (1991–2000)

During the 1990s, innovation focused on the integration of antifungal agents and the commercial availability of preformulated selective media, addressing the growing need for standardized, safer, and faster laboratory workflows (Table 4). In 1992, Stern et al. [60] developed the Campy-Cefex Agar (CCA), combining antimicrobials to suppress fungal and Gram-positive contaminants in poultry samples. Subsequently, Aspinall et al. (1993) [61] introduced the Cefoperazone Amphotericin B Teicoplanin (CAT) medium, incorporating amphotericin B and teicoplanin, further enhancing selectivity for thermophilic *Campylobacter* species, including *C. upsaliensis*. These refinements significantly improved the recovery of target isolates in mixed cultures.

The decade culminated with Jeffrey et al. (2000) [62], who designed a semi-solid aerobic medium containing rifampicin, cephalothin, and amphotericin B, allowing for *Campylobacter* recovery under non-microaerophilic conditions. This innovation anticipated the emergence of field-adapted and low-oxygen-tolerant media, bridging traditional laboratory culture and environmental surveillance methodologies.

### 4.6. Modern Complex and Enrichment Media (2001–2024)

The twenty-first century has witnessed unprecedented diversification in *Campylobacter* growth media, driven by technological advances and the demand for rapid, selective, and standardized detection in food safety and clinical diagnostics (Table 5). Line (2001) [64] introduced the Campy-Line Agar (CLA) and its blood-supplemented counterpart (CLBA), which integrated α-ketoglutaric acid, hemin, and 2,3,5-triphenyltetrazolium chloride (TTC) to allow visual differentiation of colonies and enhance detection sensitivity. Shortly thereafter, Oyarzabal et al. (2005) [65] and Thomas et al. (2005) [66] improved direct enumeration from poultry carcasses using formulations such as modified Campy-Cefex Agar (mCCA) and AAV medium, which incorporated amphotericin B, aztreonam, and vancomycin to reduce background contamination.

In 2006, the international standardization of *Campylobacter* culture methods was formalized through ISO 10272-1:2017 [58] and 10272-2:2017 [59], which established Bolton broth (for enrichment) and mCCDA (for selective isolation) as reference media. Around the same time, bioMérieux commercialized CampyFood Agar, facilitating consistent results and widespread adoption in routine testing.

Subsequent developments targeted antimicrobial resistance and recovery efficiency. Between 2012 and 2018, Chon et al. [67,68,69,70] produced a series of optimized formulations—including P-mCCDA, C-mCCDA, Modified Karmali, and Tz-Bolton broth—by supplementing traditional recipes with high concentrations of polymyxin B, potassium clavulanate, and tazobactam, improving detection of β-lactam-resistant isolates. Meanwhile, Yoo et al. (2014) [71] developed BRS agar, and Teramura et al. (2015) [72] introduced the first chromogenic medium (CM-HT) for *Campylobacter* spp., enabling rapid color-based identification.

**Figure 3:**
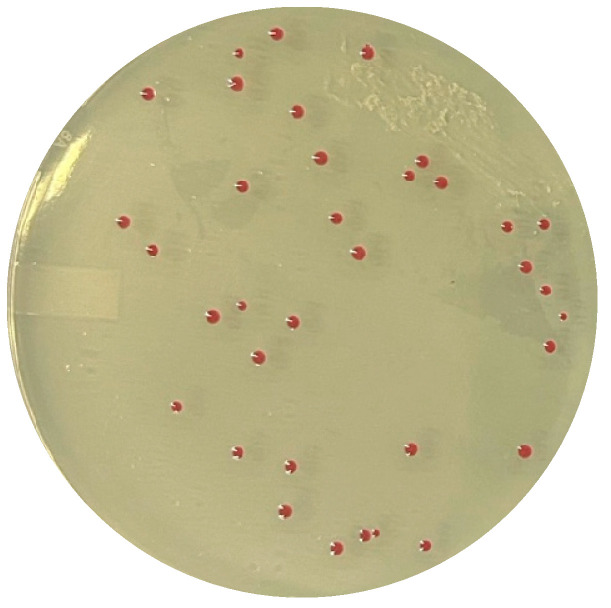
*Campylobacter jejuni* on CHROMagar™ *Campylobacter* (authors’ own photograph).

Building on this concept, CHROMagar later commercialized CHROMagar™ *Campylobacter* (Figure 3), a selective chromogenic medium designed for the routine detection and enumeration of thermotolerant *Campylobacter* spp. in food, clinical and environmental samples. Although the exact year of its initial formulation is not publicly documented, its widespread adoption in the late 2010s reflects the consolidation of chromogenic technology as a standardized, user-friendly alternative to traditional charcoal- or blood-based media.

In the 2020s, innovation has emphasized metabolic optimization and sustainability. Ha et al. (2021) [73] formulated CSA-S50, a selective agar enriched with L-serine, improving energy metabolism and recovery rates for *C. jejuni*.

Most recently, Levican and Hinton (2022) [74] presented CAMPYAIR, the first aerobically cultivable selective medium, containing soluble starch, sodium deoxycholate, and TTC, enabling *Campylobacter* spp. growth in ambient oxygen conditions.

These modern formulations embody the fusion of classical microbiological principles with molecular-era innovation, advancing toward intelligent, adaptable, and eco-efficient culture systems capable of supporting both traditional and high-throughput applications [75].

**Table 5:** Selective enrichment broths and agar-based media, including chromogenic formulations, developed for the isolation of *Campylobacter* spp., detailing basal media and selective supplements, between 2001 and 2024.

	Name	Basal Medium	Supplements (Per Liter)	Refs.
Broth	Bolton broth	α-ketoglutaric acid (1.0 g) Haemin (0.01 g) Lactalbumin hydrolysate (5.0 g) Meat peptone (10.0 g) Sodium carbonate (0.6 g) Sodium chloride (5.0 g) Sodium pyruvate (0.5 g) Sodium metabisulphite (0.5 g) Yeast extract (5.0 g)	Amphotericin B (0.01 g) Lysed horse blood (50 mL) Polymyxin B sulfate (5000 UI) Rifampicin (0.01 g) Trimethoprim lactate salt (0.01 g)	[58]
	Tz-Bolton broth	Same formulation as in Bolton broth	Same supplements as in Bolton broth Tazobactam (0.004 g)	[70]
	-	Nutrient broth No. 2	FBP supplement (0.25 g) Laked horse blood (50 mL)	[73]
	Food Pathogen Enrichment broth (FPE)	Dipotassium phosphate (0.8 g) Hemin (0.03 g) L-cystine·HCL (0.6 g) Meat extract (10.0 g) Sodium bisulfite (0.5 g) Sodium pyruvate (1.0 g) Soybean–casein digest (30.0 g)	Polymyxin B (0.0005 g) Trimethoprim (0.01 g) Cycloheximide (0.1 g) Rifampicin (0.01 g)	[76]
	R-Bolton broth	Same formulation as in Bolton broth	Same supplements as in Bolton broth Rifampin (0.0125 g)	[77]
	C-Bolton broth	Same formulation as in Bolton broth	Same supplements as in Bolton broth Potassium clavulanate (0.002 g)	[78]
Agar	Campy-Line agar (CLA)	Brucella agar (43.0 g) Hemin (0.01 g) Ferrous sulfate (0.5 g) Sodium bisulfite (0.2 g) Sodium carbonate (0.6 g) Sodium pyruvate (0.5 g) Triphenyl tetrazolium chloride (0.2 g) α-Ketoglutaric acid (1.0 g)	2,3,5-triphenyltetrazolium chloride solution (TCC) (200 ppm) Cefoperazone (0.033 g) Cycloheximide (0.1 g) Polymyxin B sulfate (0.00035 g) Trimethoprim (0.005 g) Vancomycin (0.01 g)	[64]
	Campy-Line Blood agar (CLBA)	Brucella agar (43.0 g) Ferrous sulfate (0.5 g) Sodium bisulfite (0.2 g) Pyruvic acid (0.5 g)	Lysed horse blood (50 g) Cefoperazone (0.033 g) Cycloheximide (0.1 g) Polymyxin B sulfate (0.00035 g) TCC (200 ppm) Trimethoprim (0.005 g) Vancomycin (0.01 g)	[64]
	modified Campy-Cefex Agar (mCCA)	Brucella agar (44.0 g) Ferrous sulfate (0.5 g) Sodium bisulfite (0.2 g) Sodium pyruvate (0.5 g)	Amphotericin B (0.002 g) Lysed horse blood (50 g) Sodium cefoperazone (0.033 g)	[65]
	AAV medium	*Campylobacter* Charcoal Base: peptones (10.0 g); meat extract (5.0 g); sodium chloride (5.0 g); activated charcoal (4.0 g); agar (12.0–15.0 g)	Amphotericin (0.01 g) Aztreonam (0.01 g) Vancomycin (0.01 g)	[66]
	mCCDA	Same formulation as in mCCDA (Table 3)	Same supplements as in mCCDA (Table 3) Amphotericin B (0.010 g)	[58,59]
	CampyFood Agar (CAMPY agar)	Brucella agar base (43.0 g) Ferrous sulfate (0.25 g) Sodium metabisulfite (0.25 g) Sodium pyruvate (0.25 g)	Amphotericin B (0.002 g) Lysed horse blood (70 mL) Polymyxin B sulfate (0.001 g) Trimethoprim (0.010 g) Vancomycin (0.010 g)	
	P-mCCDA	Same formulation as in mCCDA (Table 3)	Same supplements as in mCCDA (Table 3) Amphotericin B (0.002 g) Polymyxin B (100 IU)	[67]
	C-mCCDA	Same formulation as in mCCDA (Table 3)	Same formulation as in mCCDA (Table 3) Potassium clavulanate (0.0005 g)	[68]
	Modified Karmali	Same formulation as in Karmali (Table 3) Potassium clavulanate (0.5 g)	Same formulation as in Karmali (Table 3)	[69]
	BRS agar	Nutrient Broth No. 2 (Oxoid) 2% of agar 0.4% Bacteriological charcoal 0.025% (FeSO_4_-7H_2_O) 0.025% Sodium pyruvate	Rifampicin (0.010 g) Sulfamethoxazole (0.05 g)	[71]
	CM-HT	Agar (15.0 g) Ferrous sulfate (0.25 g) Proteose peptone no. 3 (10.0 g) Sodium chloride (5.0 g) Sodium deoxycholate (1.0 g) Sodium pyruvate (0.25 g) Tryptone (10.0 g) Yeast extract (2.5 g)	Amphotericin (0.002 g) Sodium cefoperazone (0.032 g) Sodium cefoxitin (0.004 g) Tetrazolium violet (0.010 g) Vancomycin hydrochloride (0.010 g)	[72]
	CHROMagar™ *Campylobacter*	Agar (15.0 g) Peptone and yeast extract (25.0 g) Salts (9.0 g)	Chromogenic and selective mix containing antibiotics (2.2 g)	
	*Campylobacter* Selective Agar (CSA) C8	Same formulation as in mCCDA Catalase (0.008 g)	Same supplements as in mCCDA	[73]
	CAMPYAIR	Beef extract (50.0 g) Tryptone (10.0 g) Sodium lactate (1.8 g) Sodium bicarbonate (1.5 g) Agar-agar (15.0 g) Soluble starch (10.0 g)	Defibrinated sheep blood (100 g) 2,3,5-triphenyltetrazolium chloride (TTC) (0.2 g)	[74]
	A-mCCDA	Same formulation as in mCCDA	Same supplements as in mCCDA Avibactam (0.0000625 g)	[79]

Across the evolution of *Campylobacter* culture media, antibiotic supplementation has played a central role in shaping selectivity and recovery efficiency. Beyond their selective role, the composition of antibiotic supplements in *Campylobacter* culture media has evolved in direct response to the progressive emergence of antibiotic resistance among competing background microbiota in clinical, food and environmental matrices. As resistance to commonly used agents such as polymyxins, trimethoprim, quinolones and macrolides increased among *Enterobacteriaceae*, *Pseudomonas* spp., *Aeromonas* spp. and Gram-positive bacteria, classical selective formulations required continuous revision to preserve suppression of competing flora without compromising *Campylobacter* recovery. It should also be acknowledged that the use of antibiotic supplements represents a potential methodological bias, as *Campylobacter* strains susceptible to the antibiotics included in selective media may be partially or completely inhibited, leading to an underestimation of their occurrence in tested samples.

This dynamic interplay between selective pressure and background flora adaptation is clearly reflected in the historical transition from early blood-based media containing relatively simple antibiotic combinations (e.g., vancomycin–polymyxin–trimethoprim in Skirrow agar) to progressively more complex formulations incorporating cefoperazone, rifampicin, aztreonam, teicoplanin and antifungal agents. More recently, the inclusion of β-lactamase inhibitors in modified mCCDA and Bolton-derived formulations further exemplifies how increasing multidrug resistance among competing microorganisms has driven iterative media redesign.

Consequently, the evolution of *Campylobacter* selective media has been shaped not only by the need to enhance selectivity, but also by the necessity to adapt to changing resistance landscapes in accompanying microbiota, ensuring robust recovery of *C. jejuni* and *C. coli* from increasingly complex and environmentally challenging matrices. This trajectory aligns with a broader One Health perspective, highlighting the influence of antibiotic use and resistance dissemination along the food chain and in the environment on diagnostic culture methodologies. Table 6 summarizes the main antibiotics employed, their biological targets, typical concentration ranges, representative media, and historical period of use.

## 5. Overview, Challenges, and Future Directions

### 5.1. Technical and Diagnostic Impact

**Figure 4:**
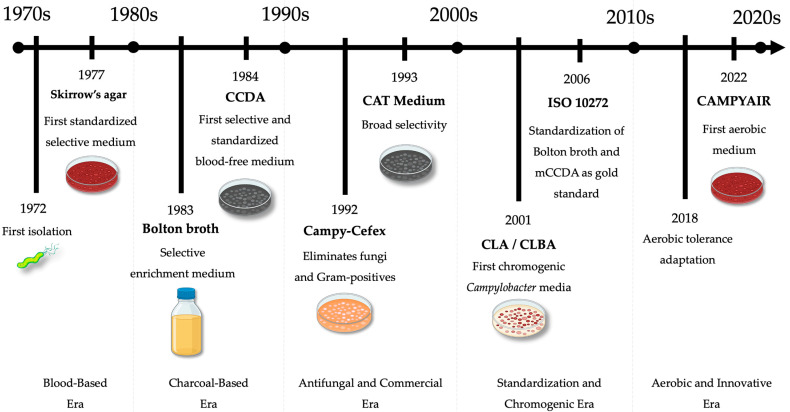
Chronological progression of *Campylobacter* media development.

Over five decades, the evolution of *Campylobacter* growth media has transformed the landscape of clinical and food microbiology (Figure 4). The Historical and contemporary culture media used for the isolation, enrichment, and enumeration of *Campylobacter* spp., including basal compositions, selective supplements, incubation time, temperature and atmospheric conditions era summarized in Appendix A (Table A1).

The introduction of blood-free, charcoal-based formulations in the 1980s represented a pivotal step toward reproducibility and accessibility. These media, exemplified by Bolton’s CCDA and Hutchinson’s mCCDA, addressed major limitations of earlier blood-containing systems, such as lot-to-lot variability and interference from heme compounds

The adoption of mCCDA in the ISO 10272-1:2017 [58] and ISO 10272-2:2017 [59] standards further cemented its role as a global benchmark for *Campylobacter* isolation in both clinical and food matrices. In addition, the Bacteriological Analytical Manual (BAM) [63] of the U.S. Food and Drug Administration specifically states that *Campylobacter* isolation should be performed using one of two selective agars: Abeyta–Hunt–Bark (AHB) [64] agar or modified Campy blood-free agar (mCCDA) [58,59], thereby formally recognizing both media as suitable options for the isolation of *Campylobacter* spp. in food microbiological analysis.

The refinement of enrichment broths, including Bolton broth and its derivatives (R-, C-, and Tz-Bolton), has also been crucial. These formulations enhanced recovery from low-level or stressed cells, enabling reliable detection even in complex samples such as poultry rinses, dairy products, and environmental water.

Collectively, the integration of selective agents such as cefoperazone, vancomycin, and trimethoprim standardized selectivity across laboratories and reduced false negatives caused by competing flora.

Furthermore, the development of antifungal and β-lactamase–inhibitor-supplemented media during the 1990s and 2000s (e.g., CAT, AAV, P-mCCDA, and C-mCCDA) addressed emerging issues of antimicrobial resistance and contamination, aligning culture performance with evolving microbial ecology.

Commercial production by companies such as bioMérieux facilitated widespread access to standardized formulations, enabling harmonized monitoring by EFSA, FDA, and WHO surveillance programs.

Decades of methodological development have resulted in a wide range of selective media for the isolation of *Campylobacter* spp. However, comparative studies consistently demonstrate that no single medium ensures optimal recovery across all sample matrices and species. In the present evaluation, all media complied with ISO 11133:2014 [80], productivity requirements (PR ≥ 0.5) and showed comparable specificity based on typical *Campylobacter* colony morphology followed by mandatory confirmation. Consequently, the main differences between media relate to their degree of selectivity and the trade-off between suppression of competing flora and recovery of stressed or less dominant species, as summarized in Table 7. Qualitative performance categories (Low, Moderate, High, Very high) were assigned based on consistent comparative trends reported across multiple studies, including relative recovery rates, detection frequencies, background flora suppression, and reference status in standardized protocols, rather than on absolute quantitative thresholds.

Charcoal-based, blood-free media such as mCCDA are widely adopted in food safety and surveillance due to their high selectivity and operational simplicity. Nevertheless, several studies have shown that cefoperazone-containing media may underrepresent non-*jejuni*/*coli* species, contributing to a recognized recovery bias. In contrast, blood-based media, including Preston agar, provide effective suppression of background flora and may support broader species recovery, albeit with higher costs and greater logistical complexity.

Beyond the choice of solid medium, methodological parameters such as direct plating versus selective enrichment, incubation temperature and atmospheric conditions further influence recovery outcomes. Direct plating preserves quantitative information and species diversity but is less sensitive at low contamination levels, whereas selective enrichment increases detection sensitivity at the expense of representativeness. Together, these considerations highlight that culture-based diagnostics for *Campylobacter* continue to evolve, with ongoing efforts to balance selectivity, sensitivity and practical applicability.

### 5.2. Limitations and Persistent Challenges

Despite major progress, culture-based detection of *Campylobacter* spp. remains challenging due to their fragile physiology and microaerophilic dependency. Traditional media still require controlled atmospheres (typically 5% O_2_, 10% CO_2_, 85% N_2_) and precise temperature regulation (41–43 °C), limiting their use in decentralized or resource-poor settings.

A recurrent limitation highlighted across comparative studies is the historical optimization of selective media for *C. jejuni* and *C. coli*. Antibiotic supplements, incubation at 42 °C and selective atmospheres systematically disadvantage non-thermotolerant and emerging *Campylobacter* species, including *C. lari*, *C. upsaliensis*, *C. fetus* and *C. hepaticus*. Recent species-focused studies demonstrate that commonly used media may fail entirely for these organisms unless formulations, supplements and incubation parameters are specifically adapted. These findings indicate that culture-based surveillance data should be interpreted with caution, as apparent epidemiological dominance may partially reflect methodological bias rather than true prevalence [93].

Additionally, sub-lethally injured or viable but non-culturable (VBNC) cells may evade recovery even on optimized media, leading to underestimation of prevalence in environmental or processed food samples [94]. VBNC *Campylobacter* cells are of particular concern in food safety and public health, as they may retain pathogenic potential while escaping detection by conventional culture-based methods. This can result in underestimation of contamination levels in foods, especially in matrices subjected to stress conditions such as refrigeration, freezing, drying, or exposure to sanitizers, and complicates quantitative risk assessment and epidemiological surveillance [95]. Furthermore, VBNC cells may resuscitate under favorable conditions along the food chain or within the host, highlighting their potential role in foodborne transmission [96]. The persistence of VBNC cells therefore reinforces the need for integrated detection strategies that combine optimized culture media with molecular approaches, rather than viewing these methodologies as mutually exclusive [94].

Moreover, the continued reliance on antibiotic supplementation, while essential for selectivity, raises ecological and biosafety concerns, particularly regarding the disposal of antimicrobial-containing waste [97].

### 5.3. Emerging Trends and Future Directions

Contemporary research is steering *Campylobacter* culture toward closer integration with molecular and omics-based methods. Several promising trends are evident: aerobic and oxygen-tolerant media enable simplified incubation without the need for microaerophilic systems, facilitating field applications and automation [92]. In addition, chromogenic and differential systems enhance colony identification and allow semi-quantitative enumeration, reducing reliance on confirmatory biochemical testing [72].

Together, these developments are transforming *Campylobacter* culture from a purely diagnostic tool into a broader research platform that increasingly complements molecular detection approaches. Despite major advances in polymerase chain reaction (PCR) and metagenomics, culture-based methods remain indispensable and are expected to retain a central role in future surveillance and research frameworks [98]. The recovery of viable isolates is essential for strain-level sequencing, outbreak investigation, source attribution, and the study of *Campylobacter* population structure and evolutionary dynamics over time [99]. In addition, culture-based isolation enables antimicrobial susceptibility testing and phenotypic characterization, while providing the reference material required to validate and contextualize molecular findings [99]. By contrast, non-culture-based approaches cannot reliably distinguish viable from non-viable or injured cells, limiting their applicability for quantitative risk assessment, regulatory decision-making, and long-term epidemiological surveillance.

In addition to these general trends, recent studies have proposed concrete modifications to culture media composition and design. These include aerobic selective formulations incorporating antioxidant components and adjusted antibiotic combinations to support *Campylobacter* growth under atmospheric conditions, as well as revised selective formulations that reduce inhibition of non-*jejuni*/*coli* species by modifying or limiting specific antibiotics [74]. Furthermore, studies have demonstrated that recovery of underrepresented *Campylobacter* species, such as *C. fetus* and *C. hepaticus*, often requires tailored supplementation and incubation parameters, as standard selective media are frequently inhibitory to these organisms [100,101].

Beyond incremental modifications of existing formulations, future development of *Campylobacter* culture media may benefit from conceptual shifts in design. One emerging direction is the use of modular or purpose-driven media systems, in which a common basal formulation is combined with adjustable selective and supplement modules tailored to the sample matrix and diagnostic objective, rather than relying on a single universal medium. Such an approach could help mitigate the trade-offs between selectivity and species diversity that characterize current formulations.

In parallel, there is growing interest in culture strategies that incorporate a preliminary recovery or resuscitation phase for sub-lethally injured or VBNC (viable but non-culturable) *Campylobacter* cells prior to exposure to selective pressure [94]. Media designs that transiently reduce oxidative and antibiotic stress may improve recovery of stressed but viable cells, with important implications for food safety surveillance and public health risk assessment.

## 6. Conclusions

The historical trajectory of *Campylobacter* spp. culture media—spanning from thioglycolate agar from Dekeyser’s (1972) [33] to CAMPYAIR from Levican and Hinton (2022) [74]—reflects an ongoing pursuit of precision, efficiency, and adaptability. Each generation of media has addressed the scientific challenges of its era: from isolation feasibility, through selectivity and standardization to modern compatibility with high-throughput technologies.

Today, the focus shifts toward multifunctional media that are not only selective and sensitive but also ecologically responsible and digitally integrable. Future breakthroughs are likely to emerge from computational design, metabolomic mapping, and synthetic biology, yielding next-generation media capable of dynamically adapting to pathogen physiology and environmental conditions.

In essence, the evolution of *Campylobacter* growth media encapsulates the broader story of microbiology itself—transforming from art to precision science, from isolation to innovation. By providing a chronological and mechanistic synthesis of *Campylobacter* culture media development, this review fulfills its objective of contextualizing past innovations while identifying future directions for diagnostic and food safety applications.

By integrating strain-specific genomic sequencing with artificial intelligence–driven modeling, next-generation culture media might be developed to enhance selective growth and recovery.

## Figures and Tables

**Table 1:** Early culture media used for the isolation of *Campylobacter* spp. (1968–1974).

	Name	Basal Medium (Per Liter)	Supplements (Per Liter)	Refs.
Broth		Fluid Thioglycolate Agar Medium (FTM-A): agar (0.75 g); casein digest (15 g); yeast extract (5 g); glucose (5 g); sodium chloride (2.5 g)	Actidione (0.5 g) Bacitracin (25 IU) Blood Novobiocin (0.05 g) Polymyxin B sulphate (10 IU)	[33]
	Butzler medium (BU)	FTM-A	Same supplements as in [34] Defibrinated ovine blood (150 g)	[40]
Agar	VA-10-B	Agar (20 g) Brucella broth (28 g): pancreatic digest of casein (10 g); peptic digest of animal tissue (10 g); yeast extract (2 g); dextrose (1 g) Sodium chloride (NaCl) (5 g) Ferrous sulfate (0.05 g) Magnesium chloride (1 g) Sodium succinate (2 g) Yeast extract (5 g)	Defibrinated bovine blood (100 g)	[35]
	VA-10-H	Same formulation as in VA-10-B	Alkaline hematin (20 mg) FBP (Ferrous–Bisulfite–Pyruvate) supplement: ferrous sulfate (0.25 g); sodium bisulfite (0.25 g); sodium pyruvate (0.25 g)	[35]

**Table 3:** Selective media developed for the isolation of *Campylobacter* spp. between 1983 and 1990 were predominantly charcoal-based and blood-free media.

	Name	Basal Medium (Per Liter)	Supplements (Per Liter)	Refs.
Broth	semisolid blood-free selective motility (SSM)	Agar (4.0 g) Mueller-Hinton broth: beef extract (2.0 g); acid hydrolysate of casein (17.5 g); starch (1.5 g)	Cefoperazone (0.03 mg) Trimethoprim (0.05 mg)	[46]
Agar	Bolton agar	Same formulation as in Preston agar (Table 2)	CFP supplement: bacteriological charcoal (4 g); ferrous sulphate (0.25 g); sodium pyruvate (0.25 g)	[56]
	Charcoal Cefoperazone Deoxycholate Agar (CCDA)	Agar (12 g) Casein enzymic hydrolysate (3 g) Charcoal (bacteriological) (4 g) Ferrous sulfate (0.25 g) Meat extract (10 g) Peptic digest of animal tissue (10 g) Sodium chloride (5 g) Sodium deoxycholate (1 g) Sodium pyruvate (0.25 g)	Cephazolin (0.010 g)	[43]
	modified CCDA (mCCDA)	Same formulation as in CCDA (Table 3)	Cefoperazone (0.032 g)	[57]
	blood-free, charcoal-based selective medium (Karmali)	Activated charcoal (4 g) Columbia agar base Hematin (0.032 g) Sodium pyruvate (0.1 g)	Cefoperazone (0.032 g) Cycloheximide (0.1 g) Vancomycin (0.02 mg)	[44]

**Table 4:** Selective media developed for *Campylobacter* spp. between 1991 and 2000 predominantly incorporated antifungal agents and refined antibiotic combinations.

	Name	Basal Medium	Supplements (Per Liter)	Refs.
Agar	Campy-Cefex Agar (CCA)	Brucella agar (44 g) Ferrous sulphate (0.5 g) Sodium bisulfite (0.2 g) Sodium pyruvate (0.5 g)	Lysed horse blood (50 g) Sodium cefoperazone (0.033 g) Sodium cycloheximide (0.2 g)	[60]
	CAT	Same formulation as in mCCDA (Table 3)	Amphotericin (0.010 g) Cefoperazone (0.008 g) Teicoplanin (0.004 g)	[61]
		Agar (0.8 g) Beef heart for infusion (2.2 g) Monopotassium phosphate (2.5 g) Proteose Peptone (10.0 g) Thioglycolate medium without indicator (29.0 g)	Antimycotic amphotericin B (2.0 g) Cephalothin (15.0 g) Polymyxin B (0.3 g) Rifampin (5.3 g) Trimethoprim (5.0 g)	[62]
	Abeyta, Hunt & Bark (AHB)	Brucella agar base (43.0 g)	Lysed horse blood (50 mL) Cefoperazone (0.033 g) Vancomycin (0.010 g) Amphotericin B (0.002 g)	[63]

**Table 6:** Antibiotics and antifungal agents used in selective *Campylobacter* culture media.

Antibiotic	Function	Typical Concentration Range	Media Examples	Period
Cefoperazone	Gram-negative selection	8–33 mg/L	mCCDA, CAT, CCA	1980s–2024
Polymyxin B	Gram-negative outer membrane	2.5–100,000 IU/L	Skirrow, Bolton, P-mCCDA	1977–2024
Trimethoprim	*Enterobacteriaceae* inhibition	5–50 mg/L	Skirrow, Preston, FPE	1977–2024
Vancomycin	Gram-positive inhibition	10–20 mg/L	Skirrow, CAT	1977–1990s
Rifampicin	Gram-positive and Gram-negative background flora suppression	5–20 mg/L	Bolton broth Preston agar Preston broth FPE broth BRS agar	1980s–2024
Teicoplanin	Gram-positive (vancomycin-resistant strains)	5–10 mg/L	CAT	1990s–2024
β-lactamase inhibitors (e.g., aztreonam, clavulanate)	Suppression of β-lactamase–producing competitors (ESBL context)	Variable (supplement-dependent)	AAV medium C-Bolton C-mCCDA Modified Karmali	2000s–2024
Amphotericin B/Cycloheximide	Antifungal	2–200 mg/L	CCA, CAT, CM-HT	1990s–2024

**Table 7:** Comparative summary of commonly used culture media for *Campylobacter* spp., including matrix applicability, performance trends, recovery bias, strengths, and limitations.

Culture Medium	Sensitivity	Selectivity	Relative LOD	Recovery Bias	Main Strengths	Main Limitations	Refs.
Skirrow agar	Moderate	Low–Moderate Low-concentration antibiotics (vancomycin, polymyxin B, trimethoprim) Incomplete inhibition of *Enterobacteriaceae* and Gram-positive bacteria; substantial background flora growth	High	Low selectivity bias	Supports recovery of stressed or antibiotic-sensitive strains	Poor inhibition of background flora; unsuitable for heavily contaminated samples	[81,82,83]
Butzler agar	Moderate	Moderate Moderate antibiotic combination (cefazolin, *coli*stin, bacitracin) Partial inhibition of Gram-positive bacteria; *Enterobacteriaceae* may persist in complex matrices	High	Variable	Balanced formulation with broader species recovery	Insufficient selectivity in complex matrices	[84,85]
Preston agar	High	High High antibiotic concentrations (cefoperazone, rifampicin, polymyxin B) Gram-positive bacteria, *Enterobacteriaceae*, and some stressed or injured *Campylobacter* strains	Low	Possible inhibition of stressed strains	Strong suppression of competing flora; effective in contaminated samples	Strong selectivity may reduce recovery of injured cells	[84,85,86]
mCCDA	High	Very high Cefoperazone + deoxycholate provide strong suppression of intestinal flora; charcoal neutralizes toxic metabolites *Enterobacteriaceae*, Gram-positive bacteria, yeasts; underrepresentation of *Campylobacter upsaliensis*, *Campylobacter fetus*, *Campylobacter hyointestinalis*	Low	Underrepresentation of non-*jejuni*/*coli* species	High selectivity; standardized; widely adopted in food safety testing	Cefoperazone-driven species bias	[82,86,87]
Campy-Cefex agar	High	High High cefoperazone concentration combined with antifungal agents *Enterobacteriaceae*, Gram-positive bacteria; cefoperazone-sensitive *Campylobacter* strains	Low–Moderate	Possible inhibition of sensitive strains	Good colony morphology; effective for poultry matrices	Reduced recovery at low contamination levels	[88,89]
Bolton broth	Very high (after enrichment)	Moderate Antibiotic supplementation (cefoperazone, vancomycin, trimethoprim) plus selective incubation conditions Competing flora suppressed; loss of less competitive *Campylobacter* species after enrichment	Very low	Enrichment bias	Enhanced detection of low-level or stressed cells	Loss of quantitative information; species representation bias	[86,90,91]
CAMPYAIR	Moderate–High	Moderate Antibiotic-based selectivity combined with aerobic tolerance Competing aerobic bacteria; incomplete suppression of intestinal background flora	Moderate	Not fully characterized	Aerobic incubation; simplified workflow	Limited validation; reduced selectivity	[92]

LOD—Limit of detection.

## Data Availability

The original contributions presented in this study are included in the article. Further inquiries can be directed to the corresponding author(s).

## Appendix A

**Table A1:** Historical and contemporary culture media used for the isolation, enrichment, and enumeration of *Campylobacter* spp., including basal compositions, selective supplements (expressed per liter), incubation time, temperature, atmospheric conditions, and bibliographic references.

	Name	Basal Medium (Per Liter)	Supplements (Per Liter)	Time	T (°C)	Atmosphere	Refs.
Broth		Fluid Thioglycolate Agar Medium (FTM-A): agar (0.75 g); casein digest (15 g); yeast extract (5 g); glucose (5 g); sodium chloride (2.5 g)	Actidione (0.5 g) Bacitracin (25 IU) Blood Novobiocin (0.05 g) Polymyxin B sulphate (10 IU)	3 days	37 °C	Microaerophilic	[33]
	Butzler medium (BU)	FTM-A	Actidione (0.5 g) Bacitracin (25 IU) Defibrinated ovine blood (150 g) Novobiocin (0.05 g) Polymyxin B sulphate (10 IU)	3 days	25 °C. 37 °C 42 °C	Microaerophilic	[40]
	Preston broth	Nutrient broth No. 2	Actidione (0.0001 g) Polymyxin (0.005 IU) Rifampicin (0.0001 g) Saponin-lysed horse blood (50 g) Trimethoprim (0.0001 g)	48 h	43 °C	Microaerophilic	[11]
	-	Brucella broth Cysteine hydrochloride (0.1g) Sodium succinate (3 g)	Cycloheximide (0.05 g) Lysed horse blood (70 g) Polymyxin B (20,000 IU) Trimethoprim (0.005 g) Vancomycin (0.015 g)	16 to 18 h	42 °C with agitation	Microaerophilic	[50]
	semisolid blood-free selective motility (SSM)	Agar (4.0 g) Mueller-Hinton broth: beef extract (2.0 g); acid hydrolysate of casein (17.5 g); starch (1.5 g)	Cefoperazone (0.03 mg) Trimethoprim (0.05 mg)	42 h	42 °C	Microaerophilic	[46]
	Bolton broth	α-ketoglutaric acid (1.0 g) Haemin (0.01 g) Lactalbumin hydrolysate (5.0 g) Meat peptone (10.0 g) Sodium carbonate (0.6 g) Sodium chloride (5.0 g) Sodium pyruvate (0.5 g) Sodium metabisulphite (0.5 g) Yeast extract (5.0 g)	Amphotericin B (0.01 g) Lysed horse blood (50 mL) Polymyxin B sulfate (5000 UI) Rifampicin (0.01 g) Trimethoprim lactate salt (0.01 g)	48 h	41.5 °C	Microaerophilic	[58]
	Tz-Bolton broth	Bolton broth according to ISO	Bolton broth supplements according to ISO 4 μg/mL of tazobactam	48 h	42 °C	Microaerophilic	[70]
	-	Nutrient broth No. 2	FBP supplement (0.25 g) Laked horse blood (50 mL)				[71]
	Food Pathogen Enrichment (FPE) broth	Dipotassium phosphate (0.8 g) Hemin (0.03 g) L-cystine·HCL (0.6 g) Meat extract (10.0 g) Sodium bisulfite (0.5 g) Sodium pyruvate (1.0 g) Soybean–casein digest (30.0 g)	Polymyxin B (0.0005 g) Trimethoprim (0.01 g) Cycloheximide (0.1 g) Rifampicin (0.01 g)	48 h	41 °C	Microaerophilic	[76]
	R-Bolton broth	Bolton broth according to ISO	Bolton broth supplements according to ISO 12.5 mg/L rifampin	48 h	42 °C	Microaerophilic	[77]
	C-Bolton broth	Bolton broth according to ISO	Bolton broth supplements according to ISO potassium clavulanate (2 mg/L)	48 h	42 °C	Microaerophilic	[78]
Agar	VA-10-B	Agar (20 g) Brucella broth (28 g): pancreatic digest of casein (10 g); peptic digest of animal tissue (10 g); yeast extract (2 g); dextrose (1 g) Sodium chloride (NaCl) (5 g) Ferrous sulfate (0.05 g) Magnesium chloride (1 g) Sodium succinate (2 g) Yeast extract (5 g)	Defibrinated bovine blood (100 g)	24–48 h	37 °C	Microaerophilic	[35]
	VA-10-H	Agar (20 g) Brucella broth (28 g) Sodium chloride (5 g) Magnesium chloride (1 g) Sodium succinate (2 g) Yeast extract (5 g)	Alkaline hematin (20 mg) FBP (Ferrous–Bisulfite–Pyruvate) supplement: ferrous sulfate (0.25 g); sodium bisulfite (0.25 g); sodium pyruvate (0.25 g)	24–48 h	37 °C	Microaerophilic	[35]
	Skirrow medium (Campy agar)	Blood agar base: peptones (10 g); meat extract (5.0 g); sodium chloride (5.0 g); agar (12.0–15.0 g)	Lysed horse blood (50 g) Polymyxin B sulphate (25 IU) Trimetropim (0.05 g) Vancomycin (0.10 g)	2 days	43 °C	Microaerophilic	[10]
	Campy-BAP	Brucella agar base: peptones (10 g); casein enzymic hydrolysate (10 g); yeast extract (2.0 g); dextrose (1.0 g); sodium chloride (5.0 g); agar (12.0–15.0 g)	Amphotericin B (2000 g) Polymyxin B (2.5 IU) Sheep red blood-cells (100 g) Trimethoprim (0.05 g) Vancomycin (0.01 g)	48 h	42 °C	Microaerophilic	[51]
	-	Brucella agar base Sodium metabisulfite (0.025%) Sodium pyruvate (0.025%)	FBP supplement (0.25 g)	48 h	42 °C	Microaerophilic	[52]
	modified Skirrow medium (mSK)	Agar base	Bacitracin (25 IU) Horse blood (50 g) Polymyxin B sulphate (2500 IU) Trimethoprim lactate (0.05 g)	2 to 5 days	42 °C	Microaerophilic	[53]
	Bolton agar						[56]
	Campy-thio	FTM-A	Amphotericin B (0.02 g) Cephalothin (0.001 g) Polymyxin B (2.5 IU) Trimethoprim (0.05 g) Vancomycin (0.10 g)	8 h	42 °C	Microaerophilic	[54]
	modified Butzler (BU40)	FTM-A	Actidione (0.5 g) Bacitracin (25,000 IU) Cephalothin (0.15 g) *Coli*stin (40,000 IU) Defibrinated sheep blood (100 g) Novobiocin (0.05 g)	72 h	42 to 43 °C	Microaerophilic	[55]
	Preston agar	Agar (1–2%) Nutrient broth No. 2: peptone (10 g); meat extract (10 g); sodium chloride (5 g)	Actidione (0.0001 g) Polymyxin (0.005 IU) Rifampicin (0.0001 g) Saponin-lysed horse blood (50 g) Trimethoprim (0.0001 g)	48 h	43 °C	Microaerophilic	[11]
	Charcoal Cefoperazone Deoxycholate Agar (CCDA)	Agar (12 g) Casein enzymic hydrolysate (3 g) Charcoal (bacteriological) (4 g) Ferrous sulfate (0.25 g) Meat extract (10 g) Peptic digest of animal tissue (10 g) Sodium chloride (5 g) Sodium deoxycholate (1 g) Sodium pyruvate (0.25 g)	Cephazolin (0.010 g)	48 h	42 °C	Microaerophilic	[43]
	modified CCDA (mCCDA)	Agar (12 g) Casein enzymic hydrolysate (3 g) Charcoal (bacteriological) (4 g) Ferrous sulfate (0.25 g) Meat extract (10 g) Peptic digest of animal tissue (10 g) Sodium chloride (5 g) Sodium deoxycholate (1 g) Sodium pyruvate (0.25 g)	Cefoperazone (0.032 g)	48 h	42 °C	Microaerophilic	[57]
	blood-free, charcoal-based selective medium (Karmali)	Activated charcoal (4 g) Columbia agar base Hematin (0.032 g) Sodium pyruvate (0.1 g)	Cefoperazone (0.032 g) Cycloheximide (0.1 g) Vancomycin (0.02 mg)	48 h	43 °C	Microaerophilic	[44]
	Campy-Cefex Agar (CCA)	Brucella agar (44 g) Ferrous sulphate (0.5 g) Sodium bisulfite (0.2 g) Sodium pyruvate (0.5 g)	Lysed horse blood (50 g) Sodium cefoperazone (0.033 g) Sodium cycloheximide (0.2 g)	48 h	42 °C	Microaerophilic	[60]
	CAT	Agar (12 g) Casein enzymic hydrolysate (3 g) Charcoal (4 g) Ferrous sulfate (0.25 g) Meat extract (10.0 g) Peptic digest of animal tissue (10.0 g) Sodium chloride (5.0 g) Sodium deoxycholate (1.0 g) Sodium pyruvate (0.25 g)	Amphotericin (0.010 g) Cefoperazone (0.008 g) Teicoplanin (0.004 g)	48 h	42 °C	Microaerophilic	[61]
	-	Agar (0.8 g) Beef heart for infusion (2.2 g) Monopotassium phosphate (2.5 g) Proteose Peptone (10.0 g) Thioglycolate medium without indicator (29.0 g)	Antimycotic amphotericin B (2.0 g) Cephalothin (15.0 g) Polymyxin B (0.3 g) Rifampin (5.3 g) Trimethoprim (5.0 g)	48 h	37 °C	Aerobic	[62]
	Abeyta, Hunt & Bark (AHB)	Brucella agar base (43.0 g)	Lysed horse blood (50 mL) Cefoperazone (0.033 g) Vancomycin (0.010 g) Amphotericin B (0.002 g)	20–44 h	42 °C	Microaerophilic	[63]
	Campy-Line agar (CLA)	Brucella agar (43.0 g) Hemin (0.01 g) Ferrous sulfate (0.5 g) Sodium bisulfite (0.2 g) Sodium carbonate (0.6 g) Sodium pyruvate (0.5 g) Triphenyl tetrazolium chloride (0.2 g) α-Ketoglutaric acid (1.0 g)	2,3,5-triphenyltetrazolium chloride solution (TCC) (200 ppm) Cefoperazone (0.033 g) Cycloheximide (0.1 g) Polymyxin B sulfate (0.00035 g) Trimethoprim (0.005 g) Vancomycin (0.01 g)	42 h	42 °C	Microaerophilic	[64]
	Campy-Line Blood agar (CLBA)	Brucella agar (43.0 g) Ferrous sulfate (0.5 g) Sodium bisulfite (0.2 g) Pyruvic acid (0.5 g)	Lysed horse blood (50 g) Cefoperazone (0.033 g) Cycloheximide (0.1 g) Polymyxin B sulfate (0.00035 g) TCC (200 ppm) Trimethoprim (0.005 g) Vancomycin (0.01 g)	42 h	42 °C	Microaerophilic	[64]
	modified Campy-Cefex Agar (mCCA)	Brucella agar (44.0 g) Ferrous sulfate (0.5 g) Sodium bisulfite (0.2 g) Sodium pyruvate (0.5 g)	Amphotericin B (0.002 g) Lysed horse blood (50 g) Sodium cefoperazone (0.033 g)	48 h	42 °C	Microaerophilic	[65]
	AAV medium	*Campylobacter* Charcoal Base: peptones (10.0 g); meat extract (5.0 g); sodium chloride (5.0 g); activated charcoal (4.0 g); agar (12.0–15.0 g)	Amphotericin (0.01 g) Aztreonam (0.01 g) Vancomycin (0.01 g)	24 h	37 °C	Microaerophilic	[66]
	mCCDA	Activated charcoal (4.0 g) Agar (12–15 g) Casein hydrolysate (3.0 g) Ferrous sulfate (0.25 g) Meat extract (10.0 g) Peptone (10.0 g) Sodium chloride (5.0 g) Sodium deoxycholate (1.0 g) Sodium pyruvate (0.25 g)	Cefoperazone (0.032 g) Amphotericin B (0.010 g)	48 h	41.5 °C	Microaerophilic	[58,59]
	CampyFood Agar (CAMPY agar)	Brucella agar base (43.0 g) Ferrous sulfate (0.25 g) Sodium metabisulfite (0.25 g) Sodium pyruvate (0.25 g)	Amphotericin B (0.002 g) Lysed horse blood (70 mL) Polymyxin B sulfate (0.001 g) Trimethoprim (0.010 g) Vancomycin (0.010 g)	48 h	41.5 °C	Microaerophilic	
	P-mCCDA	Agar (12.0 g) Casein enzymic hydrolysate (3.0 g) Charcoal (4.0 g) Ferrous sulfate (0.25 g) Meat extract (10.0 g) Peptic digest of animal tissue (10.0 g) Sodium chloride (5.0 g) Sodium deoxycholate (1.0 g) Sodium pyruvate (0.25 g)	Amphotericin B (0.002 g) Cefoperazone (0.032 g) Polymyxin B (100 IU)	48 h	42 °C	Microaerophilic	[67]
	C-mCCDA	mCCDA base according to ISO	mCCDA supplements according to ISO Potassium clavulanate (0.0005 g)	48 h	42 °C	Microaerophilic	[68]
	Modified Karmali	Columbia agar base Activated charcoal (4.0 g) Hematin (0.032 g) Sodium pyruvate (0.1 g) Potassium clavulanate (0.5 g)	Cefoperazone (0.032 g) Cycloheximide (0.100 g) Vancomycin (0.020 g)	48 h	42 °C	Microaerophilic	[69]
	BRS agar	Nutrient Broth No. 2 (Oxoid) 2% of agar 0.4% Bacteriological charcoal 0.025% (FeSO_4_-7H_2_O) 0.025% Sodium pyruvate	Rifampicin (10 mg/L) 50 mg/L sulfamethoxazole	48 h	42 °C	Microaerophilic	[71]
	CM-HT	Agar (15.0 g) Casamino acids (3.0 g) Ferrous sulfate (0.25 g) Proteose peptone no. 3 (10.0 g) Sodium chloride (5.0 g) Sodium deoxycholate (1.0 g) Sodium pyruvate (0.25 g) Tryptone (10.0 g) Yeast extract (2.5 g)	Amphotericin (0.002 g) Sodium cefoperazone (0.032 g) Sodium cefoxitin (0.004 g) Tetrazolium violet (0.010 g) Vancomycin hydrochloride (0.010 g)	48 h	42 °C	Microaerophilic	[72]
	CHROMagar™ *Campylobacter*	Agar (15.0 g) Peptone and yeast extract (25.0 g) Salts (9.0 g)	Chromogenic and selective mix containing antibiotics (2.2 g)	48 h	42 °C	Microaerophilic	
	A-mCCDA	mCCDA base according to ISO	mCCDA supplements according to ISO Avibactam 0.0625 mg/L.	48 h	42 °C	Microaerophilic	[79]
	*Campylobacter* Selective Agar (CSA) C8	Agar (12.0 g) Casein hydrolysate (3.0 g) Catalase (0.008 g) Charcoal (4.0 g) Ferrous sulfate (0.25 g) Nutrient broth (25.0 g) Sodium deoxycholate (1.0 g)	Amphotericin B (0.01 g) Cefoperazone (0.032 g)	48 h	42 °C	Microaerophilic	[73]
	CAMPYAIR	Beef extract (50.0 g) Tryptone (10.0 g) Sodium lactate (1.8 g) Sodium bicarbonate (1.5 g) Agar-agar (15.0 g) Soluble starch (10.0 g)	Defibrinated sheep blood (100 g) 2,3,5-triphenyltetrazolium chloride (TTC) (0.2 g)	48 h	42 °C	Microaerophilic	[74]
